# Exposure to predator threat engages sympathetic nervous system outflow to skeletal muscle

**DOI:** 10.1016/j.autneu.2025.103304

**Published:** 2025-06-01

**Authors:** Erin Gorrell, Ashley M. Shemery, Leah Franks, Noor Samman, Meredith Zendlo, Emily Welch, Cassidy Ridley, Ashely Davis, Amber R. Titus, Lydia A. Heemstra, Colleen M. Novak

**Affiliations:** a School of Biomedical Sciences, Kent State University, Kent, OH 44242, United States of America; b Department of Biological Sciences, Kent State University, Kent, OH 44242, United States of America

**Keywords:** Predator odor, Thermogenesis, Brown adipose tissue, Norepinephrine turnover, Rat, Adrenergic

## Abstract

As obesity and overweight continue to be a public health burden throughout the world, approaches to increase energy expenditure (EE) are sought to reverse the energy imbalance underlying weight gain. Skeletal muscle is a promising target for enhancing energy expenditure. We have previously shown that exposure to predator odor induces skeletal muscle thermogenesis and results in increased physical activity, energy expenditure, and weight loss in rats. Prior evidence supports the importance of sympathetic nervous system (SNS) activation of skeletal muscle through β adrenergic receptors. Here, we measured norepinephrine turnover (NETO) rate to demonstrate that predator threat increases SNS outflow to skeletal muscle and brown adipose tissue (BAT), as well as some white adipose depots, in rats. We surgically excised the primary BAT depot to probe the potential contribution of BAT to muscle thermogenesis. Rats lacking interscapular BAT (iBATX) showed no discernable deficit in predator odor-induced muscle thermogenesis, but showed some differential response to the β3 adrenergic agonist CL-316,243. Altogether, this reaffirms the importance of SNS outflow to skeletal muscle in the thermogenesis elicited by predator threat.

## 1. Introduction

For long-term weight loss and maintenance of reduced weight and adiposity, it is necessary to address the underlying cause of obesity-—energy imbalance, where energy input exceeds output—and target mechanisms that reverse this imbalance. Exploration of alternative strategies to increase energy expenditure (EE) passively through heat production (Ginter and Simko, 2012; Palmer and Clegg, 2017; Poekes et al., 2015) have focused primarily on brown adipose tissue (BAT). BAT has the capacity to increase EE in rodents through non-shivering thermogenesis (Ginter and Simko, 2012; Poekes et al., 2015) driven by autonomic outflow, specifically the sympathetic nervous system (SNS) (Bahler et al., 2015; Labbe et al., 2015). BAT is abundant in small mammals due to its essential function of heat production during cold exposure, with interscapular BAT (iBAT) being the largest and most predominant contributor to non-shivering thermogenesis (Grunewald et al., 2018). BAT is much less abundant in humans, however, making up only about 0.2 % of total body mass in adults (Carpentier et al., 2018; Lidell et al., 2013), and accounting for only 7–25 kcal expenditure per day in humans (Carpentier et al., 2018; Lidell et al., 2013), calling into question its ability to significantly elevate EE in humans.

Conversely, skeletal muscle, the primary site for glucose metabolism, makes up 30–40 % of the body composition in humans (Janssen et al., 2000). In addition to EE during contraction, skeletal muscle produces heat through shivering and non-shivering thermogenesis (Periasamy et al., 2017). Compared to the neural pathways mediating BAT thermogenesis (Morrison and Madden, 2014; Munzberg et al., 2016), however, much less is known regarding the control of muscle non-shivering thermogenesis. This is particularly surprising given that skeletal muscle, which comprises a greater proportion of total body mass than BAT, is a promising target for increasing EE. We have established that skeletal muscle thermogenesis can be induced in laboratory rodents following exposure to predator (ferret) odor (Gorrell et al., 2020). This effect is accompanied by overall increased EE and enhanced weight loss (Gorrell et al., 2020; Shemery et al., 2023), suggesting a potential pathway through which skeletal muscle non-shivering thermogenesis can be harnessed to treat obesity. Evidence from pharmacological and denervation studies indicates that predator odor-induced skeletal muscle thermogenesis is mediated primarily through sympathetic neural activation of muscle beta, most likely beta-2, adrenergic receptors (β2-ARs) (Gorrell et al., 2020). However, the extent to which exposure to predator threat elevates SNS outflow in skeletal muscle has not been determined. Moreover, we have observed higher absolute temperatures in BAT compared to skeletal muscle (Gorrell et al., 2020), and the question arises whether BAT influences skeletal muscle temperature. Alternatively, exposure to predator threat could elevate SNS outflow to both BAT and skeletal muscle. Therefore, the second goal of this investigation was to determine if BAT contributes to the temperature increase in muscle seen in response to predator threat by examining whether BAT removal dampens the magnitude of muscle thermogenesis.

Here, we determined the concurrent elevation of SNS outflow to thermogenic tissues including skeletal muscle and interscapular BAT (iBAT) by measuring the rate of norepinephrine (NE) turnover (NETO), a measure of SNS outflow to target tissues (Vaughan et al., 2014). Subsequently, we examined the potential contribution of BAT to the elevated thermogenesis and EE seen in skeletal muscle by removing iBAT, the largest BAT depot in rats, where removal of iBAT is able to induce detectable physiological changes (Bartness et al., 2010b; Grunewald et al., 2018); the impact of iBAT removal was also probed using the β3-AR agonist CL-316,243. In this context, we also probed for potential sex differences in muscle thermogenesis; though previous evidence indicates that both male and female rats and mice show predator odor-induced muscle thermogenesis, with no change over the estrous cycle in rats (Gorrell et al., 2020; Watts et al., 2024), the magnitude of thermogenic induction after predator threat has yet to be directly compared between male and female rats.

## 2. Methods

### 2.1. Norepinephrine turnover (NETO)

By blocking alpha methyl-para-tyrosine (AMPT), the rate limiting step in catecholamine synthesis, tissue catecholamine levels are measured to indicate tissue SNS outflow, as detailed by Vaughan et al. (2014).

### 2.1.1. Animals

Male Sprague-Dawley rats (n = 24) purchased from Envigo were individually housed in a temperature-controlled room (24 °C ± 1 °C) on a 12/12 h light/dark cycle with lights on at 7:00 AM and light off at 7:00 PM Eastern Standard Time. Rats were given free access to both water and standard laboratory chow (5P00 Prolab RMH 3000, LabDiet, St. Louis, MO, USA). All procedures were approved by the Kent State University Animal Care and Use Committee, and aligned with the Guide for the Care and Use of Laboratory Animals (1996).

### 2.1.2. Alpha methyl-para-tyrosine (AMPT) injection and home-cage predator odor exposure

**Fig. 1.**
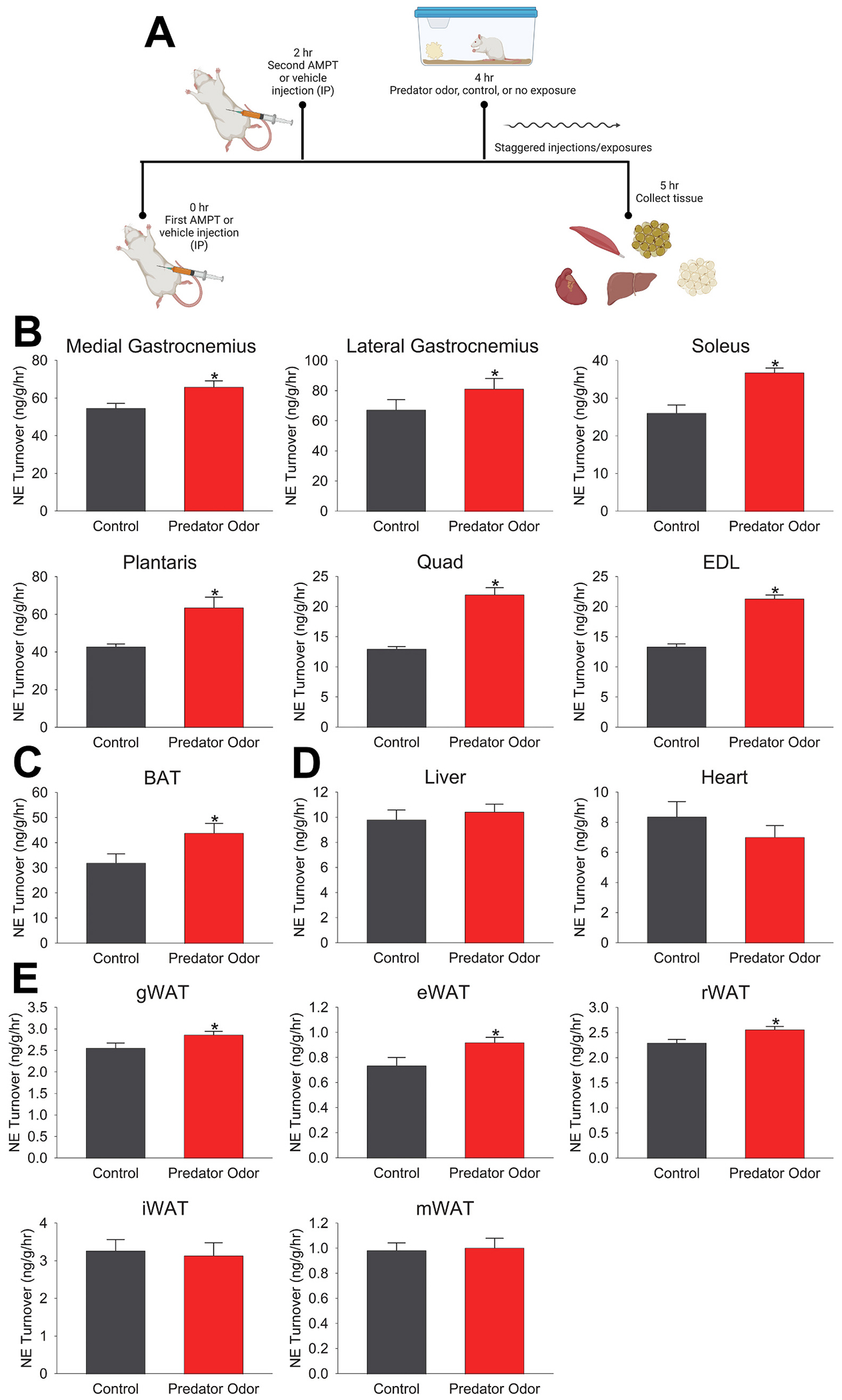
Norepinephrine turnover (NETO) revealed elevated sympathetic outflow to skeletal muscles, brown adipose tissue, and some white adipose tissue (WAT) depots. (A) Workflow for the measurement of NETO rate using injection of alpha methyl-para-tyrosine (AMPT) in rats exposed to predator odor or control odor; created with BioRender.com. (B) Exposure to predator odor significantly increased NETO in skeletal muscles: medial and lateral gastrocnemius, soleus (n = 7/exposure), plantaris, quadriceps (quad), and extensor digitorum longus (EDL). Predator odor exposure also increased NETO in brown adipose tissue (BAT) (C), but no increase was detected in liver or heart (D). (E) Moderately but significantly higher NETO was seen in some white adipose tissue (WAT) depots including gluteal (gWAT), epididymal (eWAT), and retroperitoneal (rWAT), but not inguinal (iWAT; n = 4 predator odor and 5 control-exposed) or mesenteric (mWAT). *Significantly elevated over control exposure, p < 0.05, 1-tailed *t*-test; n = 8/exposure unless otherwise noted.

All rats were habituated to handling, mild restraint to simulate injection protocol, and daily exposure to control odor in their home cages for 7 days prior to experimental odor exposure and injections. Control or predator (ferret) odors were presented by dropping a fragment (1–2″ X 2″) of a clean towel or an identical towel that had been housed with ferrets (*Mustela putorius furo*) for 2 weeks (Marshall BioResources, North Rose, NY) into the rat’s home cage. Rats were administered AMPT (125 mg/kg, I.P.; 0.9 % sterile saline vehicle, I.P.), with a second, identical injection following 2 h later; the first rat was injected starting 1 h after the start of the light phase, with subsequent rats injected at 5-min increments thereafter. During this time, predator odor or control odor towels were dropped into home cages, beginning with the first rat to receive their second AMPT injection, or no injection, and this continued in sequence with 5 min between odor exposures to allow adequate time for tissue collection. Rats received either two injections prior to predator or control odor exposure, or no injections and no odor exposure. Each rat was exposed to one of the following conditions: AMPT/predator odor (n = 8), AMPT/control odor (n = 8), or no injection/no odor (n = 8). Animals were sacrificed by rapid decapitation, without anesthesia, after 1 h of predator odor, control, or no odor exposure (Fig. 1). All experimental procedures took place in a random, counterbalanced order over 2 days.

### 2.1.3. Tissue catecholamine analysis

After euthanasia, tissues were dissected, rapidly frozen, and stored at −80 °C. Muscle tissues dissected included lateral and medial gastrocnemius muscles, soleus, plantaris, quadriceps, and extensor digitorum longus (EDL). Other tissues included iBAT, white adipose tissue (epididymal, eWAT; retroperitoneal, rWAT; mesenteric, mWAT; inguinal, iWAT; and gluteal, gWAT) as well as heart and liver.

### 2.1.4. High performance liquid chromatography (HPLC)

Tissue NE content was measured using HPLC. Catecholamine extraction was performed using a protocol adapted from Gavini et al. (2016). Briefly, tissue was weighed (0.100–0.250 g) and added to a tube containing 0.2 M perchloric acid. Internal standard 1000 ng/ml DHBA was added (100 μl), and tissue was homogenized (Fisher Scientific Sonic Dismembrator Model 100, Thermofisher Scientific, Waltham, MA) for 30 s, pulsing on/off and chilling on ice between homogenizations.

Samples were centrifuged at 4000 RCF for 15 min at 4 °C. Homogenate (600 μl) was pipetted to a new tube containing 150 mg alumina (Al2O3), and 0.5 M Tris base (pH 8.6) was added (1 ml). Samples were placed on ice and vortexed briefly every 3 min for 30 min to allow for catecholamine absorption. Samples were centrifuged at 4000 RCF for 3 min at 4 °C, and supernatant was removed and discarded. HPLC water (1 ml) was used to wash alumina twice, and samples were centrifuged at 4000 RCF for 3 min at 4 °C between each wash. Catecholamines were eluted with 0.2 M perchloric acid (300 μl), and samples were filtered through sterile syringe filters (pore size: 0.2 μm, diameter: 25 mm) and stored at −80 °C. Catecholamine standards (Thermofisher Scientific, Waltham, MA) were prepared at the following concentrations: 3.91, 7.81, 15.63, 31.25, 62.5, 125 ng/ml, and catecholamines were assayed using an HPLC system with electrochemical detection (Coulochem III), MD-TM mobile phase (Thermofisher Scientific, Waltham, MA), and a reverse phase MD 150 × 3.2 column.

### 2.1.5. NETO rate

NETO rate was calculated based on tissue NE concentration, according to the equation reported by Gavini et al. (2016) and Gavini et al. (2018)

k=(lg[NE]O−lg[NE]4)/(0.434×4)K=k[NE]OK=NETO

where:

[NE]O = vehicle

[NE]4 = predator odor or control odor.

### 2.2. iBAT excision (iBATX)

The contribution of BAT to muscle thermogenesis was examined by comparing the ability of ferret odor to provoke thermogenesis in rats with their largest BAT depot, iBAT, surgically excised, compared to rats with intact BAT. In addition to thermogenesis, we used indirect calorimetry to examine metabolic rate and locomotor activity as well as respiratory exchange ratio (RER; VCO_2_/VO_2_), where a lower RER indicates increased fat oxidation. In this context, the thermogenic induction was directly compared between female and male rats.

#### 2.2.1. Animals

Adult male and female Sprague-Dawley rats (N = 16) purchased from Envigo were individually housed as described above (Section 2.1.1).

#### 2.2.2. Surgical excision of iBAT with transponder implantation

The primary BAT depot, iBAT, was bilaterally excised to determine the role of BAT on predator odor-induced thermogenesis. Rats received iBAT excision (iBATX) or sham surgery (n = 8 iBATX, n = 4 females and n = 4 males; n = 8 sham, n = 4 females and n = 4 males) at 8 weeks of age. Rats were anesthetized with isoflurane (5 % induction, 2–3 % maintenance), the surgical site was prepared with alcohol and betadine, and an incision was made on the dorsal surface between the scapula. Ketoprofen (5 mg/kg, I.P.) was administered following the initial 5 % isoflurane induction but before incision. The iBAT pads were dissected and removed in iBATX rats or exposed but not removed in sham animals. Using a sterile syringe, epinephrine (≤0.2 cm^3^, sterile lidocaine HCl 2 % and epinephrine, 1:100,000) was dropped into the surgical site to reduce bleeding. Absorbable sutures were used to close the dorsal musculature, and subcuticular sutures were used to close the dermal incision.

**Fig. 2.**
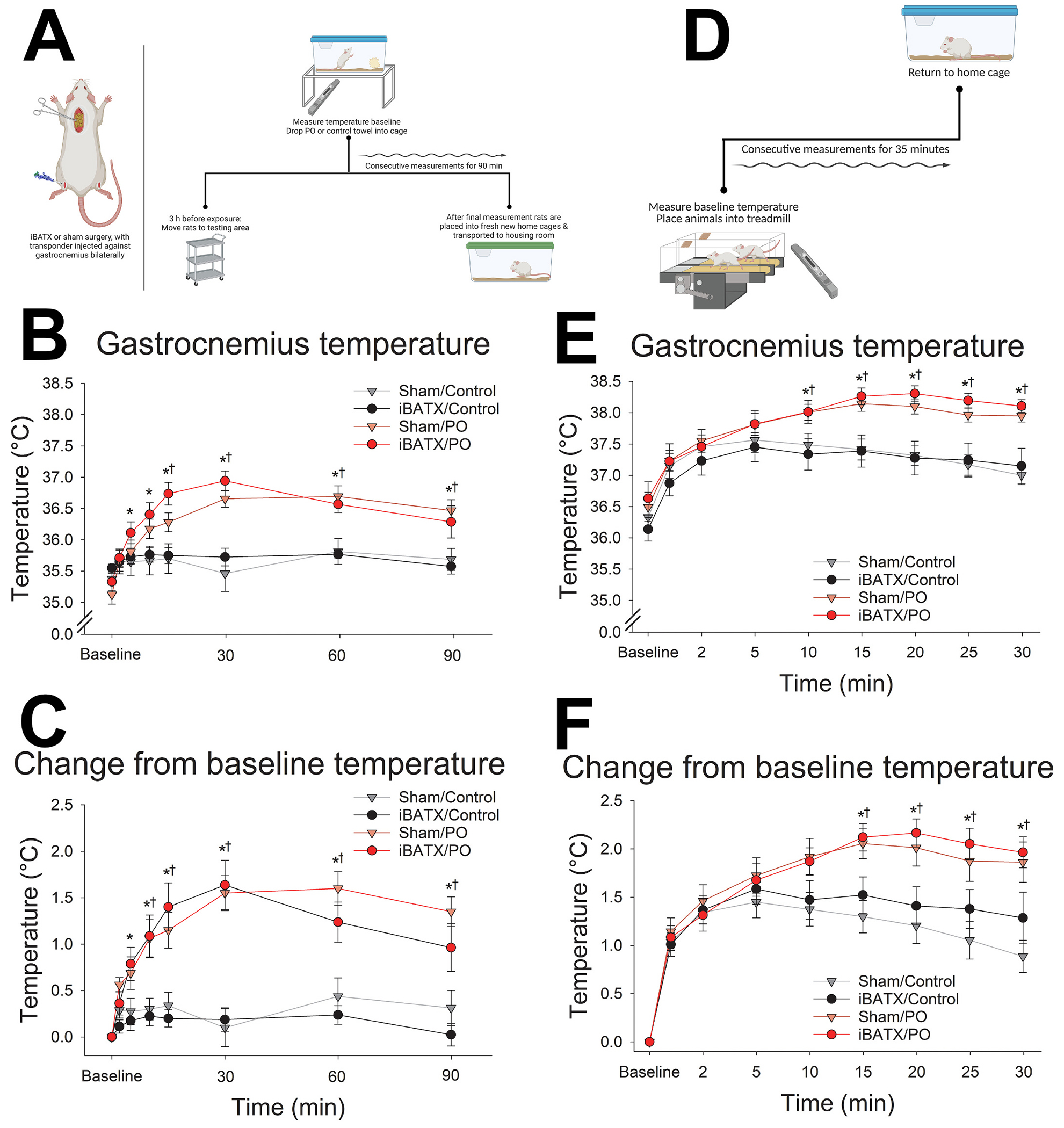
Exposure to predator threat enhanced muscle thermogenesis in rats either in the presence or absence of interscapular brown adipose tissue (iBAT). (A) Workflow for removal of iBAT (iBATX) and subsequent measurement of muscle thermogenesis; created with BioRender.com. (B) Exposure to predator threat in the form of ferret odor significantly increased muscle temperature in rats with intact brown adipose tissue (BAT) or rats that underwent removal of iBATX, with no decrement after iBATX relative to sham control. (C) Change in muscle temperature relative to baseline temperature was elevated after predator odor (PO) compared to control exposure in both sham and iBATX rats. (D) Controlled treadmill walking at 7 m/min elevated (E) muscle temperature and (F) temperature change relative to each rat’s baseline significantly more after exposure to PO compared to control odor, with no difference between rats with iBATX compared to sham controls. Significantly elevated above control-odor exposure in iBATX rats (*) and rats with intact BAT (sham, †), p < 0.05. N = 8 rats (4 male and 4 female) with each iBATX and sham.

Concurrently, temperature transponders (IPTT-300, BioMedic Data Systems, Seaford, DE, USA; calibrated range 32–43 °C) were implanted in muscle to measure changes in skeletal muscle temperature in response to predator-odor stimuli, as described previously (Gorrell et al., 2020; Shemery et al., 2023). A 2 mm incision was made in each hindlimb, and transponders (14 mm long and 2 mm in diameter) were implanted bilaterally using a pre-loaded injector (Fig. 2A). Ketoprofen (5 mg/kg, I.P.) was administered 24 h postoperatively for pain-reduction maintenance according to veterinarian recommendation, and rats were allowed to recover for 7 days.

#### 2.2.3. Home-cage predator odor-induced muscle temperature

Transponder readers (DAS-700R, 7007S, or 6007) were used to manually retrieve transponder temperature data. All rats were habituated to handling and daily exposure to control odor in their home cages for 10–14 days prior to experimental odor exposure, and control odor was presented between each experimental odor exposure. Control odor or predator odor exposure was presented by dropping a fragment (1–2″ X 2″) of a clean towel or an identical towel that had been housed with ferrets (*Mustela putorius furo*) for 2 weeks (Marshall BioResources, North Rose, NY) into the animal’s home cage. 2 h after lights-on, animals were moved from their housing room to a separate experimental room and given 3 h to acclimate; cages were topped with filters (Ancare, R20 Micro Filter Top – Rat, Bellmore, NY). 5 h after lights-on (ZT 5), baseline temperatures were measured, then stimulus or control towels were immediately presented. Temperatures were measured immediately following odor exposure at defined time points for 1.5 h (Fig. 2). Human interference was minimized by the capability of the transponder reader to measure through the cage walls, without disrupting the animals (Watts et al., 2022).

#### 2.2.4. Treadmill predator odor-induced muscle temperature

Rats were trained to walk on the treadmills prior to experimentation. All rats experienced experimental conditions in random order. Baseline temperatures were measured at 2 h after lights-on. Then, rats were placed in an enclosed treadmill set at 0° incline and a walking speed of 7 m/min with either control or predator odor towel fragments affixed to the inner wall of the treadmill housing. The treadmill protocol lasted for 35 min; muscle temperatures were measured at regular intervals (Gavini et al., 2014; Watts et al., 2022).

#### 2.2.5. Calorimetry after predator odor exposure or β3-AR agonist

**Fig. 3.**
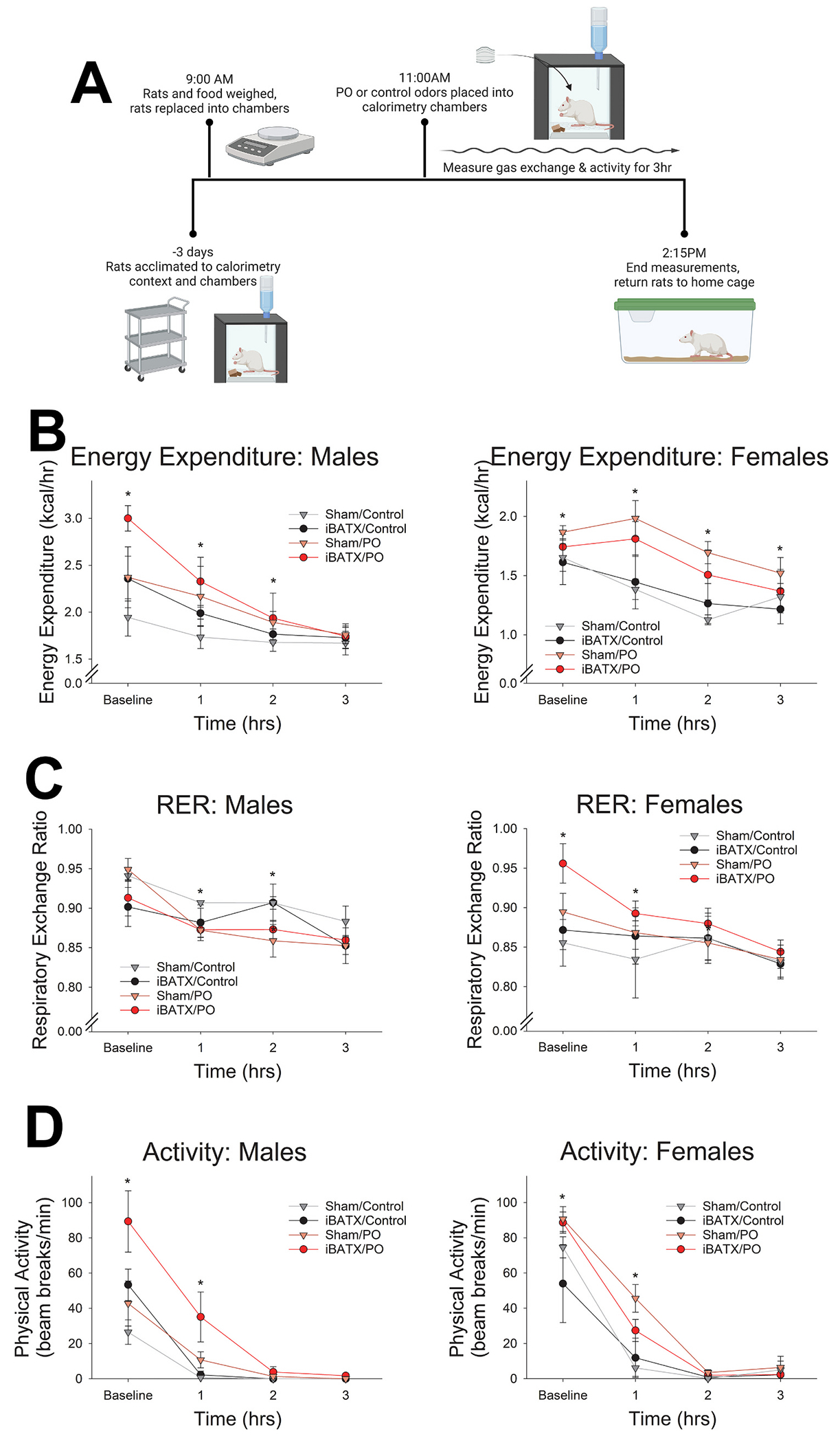
Exposure to predator threat elevated energy expenditure (EE) and physical activity in rats either in the presence or absence of interscapular brown adipose tissue (iBATX). (A) Workflow for acclimation, calorimetry, and exposure to predator odor (PO) or control odor; created with BioRender.com. (B) Both male and female rats showed significantly elevated EE after PO exposure compared to control-odor exposure. (C) Exposure to PO suppressed respiratory exchange ratio (RER; VO_2_/VCO_2_) in male, whereas female rats showed elevated RER relative to control-odor exposure. (D) Physical activity, measured as ambulatory beam-break counts, was elevated after exposure to predator odor in both male and female rats. There were no significant differences in EE or RER between rats with iBAT removal (iBATX) and sham controls. N = 8 rats (4 male and 4 female) with each iBATX and sham. *Significant change with PO compared to control exposure, p < 0.05.

Gas exchange was measured using small-animal indirect calorimetry to assess changes in caloric expenditure and substrate utilization with predator odor exposure using a 4-chamber Oxymax FAST system with infrared activity monitors (Columbus Instruments, Columbus, OH), as previously described (Gorrell et al., 2020). All calorimetry took place in a randomized, counterbalanced order over 10 days. Rats were moved to calorimetry chambers and allowed ad libitum access to food and water for three days prior to experimentation. 2 h after lights-on, rats and food were weighed and placed into calorimetry chambers. Rats were enclosed in calorimetry chambers and fresh air was supplied to each chamber at 2.79–3.0 l per minute (LPM), depending on the weight of the rat, with the same LPM within rat between tests. Sample air was measured at 0.5 LPM with reference air measured after each 30 samples; each reference measurement resulted in a 3.5-min break in experimental measurement. Physical activity was assessed using infrared beam breaks to determine beam-break counts, ambulatory counts (non-consecutive bream breaks), and vertical activity (e.g., rearing). After 120 min, control or predatorodor towels were placed in the calorimetry chambers during the reference measurement (Fig. 3A). Carbon dioxide (CO_2_) and oxygen (O_2_) gas exchange and physical activity measurements continued for 3 h.

**Fig. 4.**
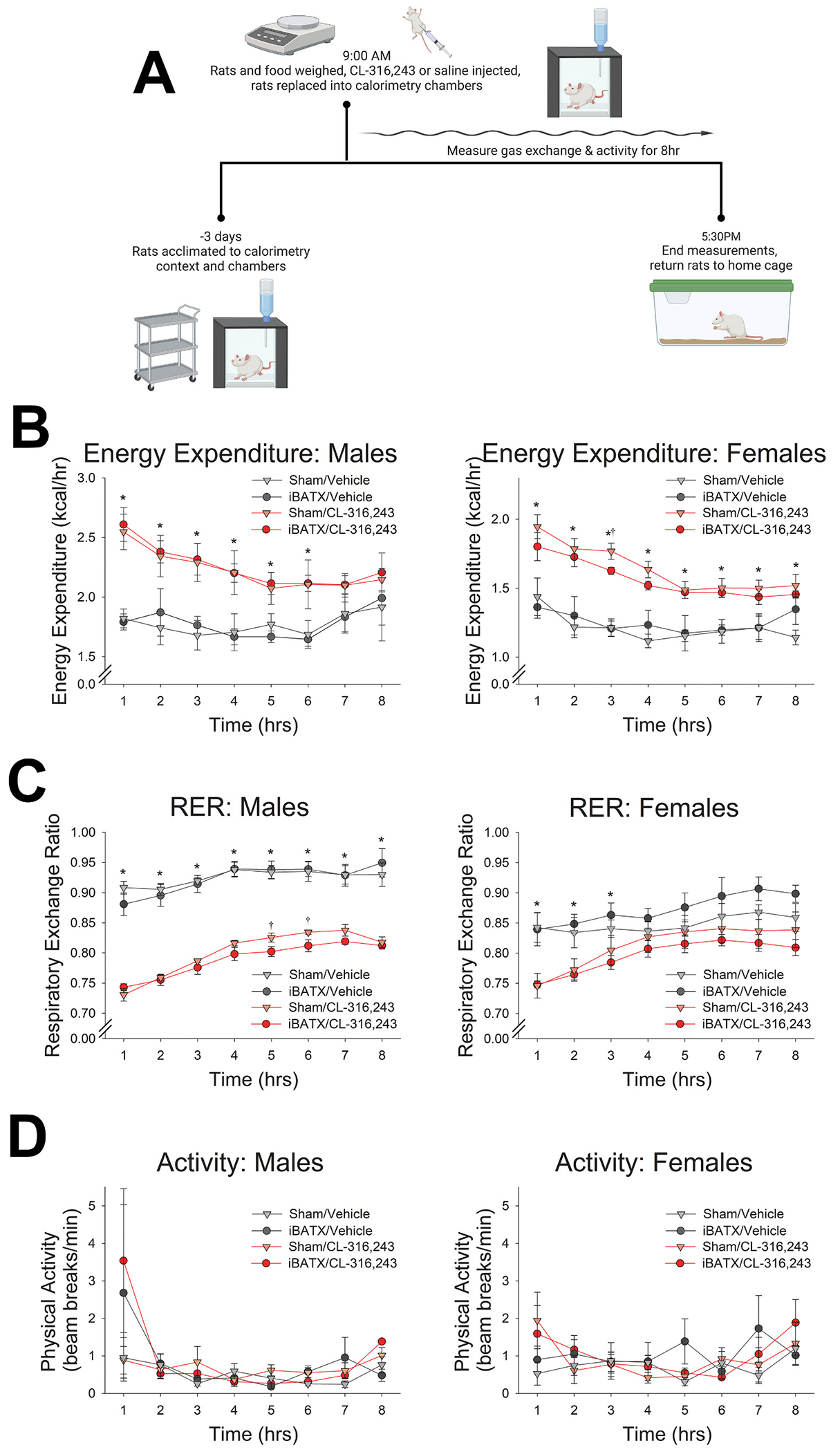
BAT excision altered the ability of the β3-AR agonist CL-316,243 to alter energy expenditure (EE) and substrate utilization. (A) Workflow illustrating treatment with the β3-AR agonist CL-316,243 (5 mg/kg) followed by measurement of gas exchange using indirect calorimetry; created with BioRender.com. (B) The β3-AR agonist CL-316,243 significantly increased EE in rats after both BAT excision (iBATX) and sham surgery, with the β3-AR agonist CL-316,243 elevating EE significantly less in female rats with iBATX compared to the sham surgery at one timepoint (3 h). (C) CL-316,243 also lowered respiratory exchange ratio (RER) in both male and female rats, with a significantly different effect on male rats with iBATX compared to sham. (D) There were no significant effects of either iBATX or CL-316,243 on ambulatory activity. *Significant effect CL-316,243 or †iBATX within timepoint, p < 0.05, error bars represent ±SEM. N = 8 rats (4 male and 4 female) with each iBATX and sham.

Calorimetry was also used to assess changes in caloric expenditure and substrate utilization following injection of the β3-AR agonist CL-316,243 (Fig. 4A). CL-316,243 is potently selective for β3-AR (Bloom et al., 1992) and does not cross the blood brain barrier (Natarajan et al., 2024; Sum et al., 1999), allowing for peripheral activation of β3-ARs to compare EE between iBATX and sham-operated rats. All testing took place as described above in a randomized, counterbalanced order over 17 days. 2 h after lights-on (ZT 2), rats and food were weighed, and rats were injected with either vehicle (0.9 % sterile saline, I.P.) or CL-316,243 (5 mg/kg, I.P.) then placed in calorimetry chambers. Gas exchange and physical activity measurements continued for 8 h.

### 2.3. Statistical analyses

Analyses were completed using IBM^®^ SPSS^®^ Statistics software version 26. Tissue NETO was compared between predator odor- and control odor-exposed rats using 1-tailed paired-samples *t*-tests. Muscle thermogenesis over time and muscle temperature change from baseline were analyzed using separate 3-way repeated-measures analyses of variance (ANOVA). Treadmill walking muscle temperature and temperature change from baseline were analyzed using 2-way repeated-measures ANOVA. Body weight comparisons were analyzed using paired-samples *t*-tests. Calorimetry measurements (energy expenditure, RER, locomotion) were analyzed using 3-way repeated measures ANOVA. Significant effects were analyzed using post-hoc paired samples t-tests.

## 3. Results

### 3.1. Predator threat increased SNS outflow to skeletal muscle as well as to BAT and some WAT depots

SNS outflow was assessed at the tissue level by measuring NETO rate (Fig. 1A). As shown in Fig. 1B, exposure to predator threat significantly increased NETO in all skeletal muscles analyzed. Predator threat also increased NETO in BAT but not liver or heart (Fig. 1C–D). For white adipose tissue, gWAT, eWAT, and rWAT showed moderate but significant increases in NETO, while iWAT and mWAT did not (Fig. 1E).

### 3.2. Surgical excision of iBAT did not significantly alter predator odor induced muscle thermogenesis

To determine if iBAT, the largest BAT depot and most predominant contributor to BAT thermogenesis (Bartness et al., 2010b; Grunewald et al., 2018; Marks et al., 2009; Saito et al., 2009), alters predator odor-induced skeletal muscle thermogenesis, iBAT was surgically excised and muscle thermogenesis was assessed (Fig. 2A). Critically, there was no significant main effect of iBATX or interactions between iBATX and odor (F(1, 14) = 0.221, p = 0.646), iBATX and time (F(7, 98) = 1.53, p = 0.165), or iBATX, odor, and time (F(7, 98) = 0.694, p = 0.568). Post-hoc comparisons of predator odor and control odor response in rats with iBATX in Fig. 2B show that predator odor exposure significantly increased muscle temperature relative to control odor beginning at 5 min (t(7) = 3.21, p = 0.015) and remaining elevated through 90 min (t(7) = 3.60, p = 0.009). The response to predator odor was similar to rats with intact iBAT (sham surgical control), which showed a significant elevation in muscle temperature starting at 15 min (t(7) = 2.57, p = 0.037) through 90 min (t(7) = 2.94, p = 0.022). There was no significant interaction between odor and sex (F(1, 12) = 2.07, p = 0.172) and no other significant sex interactions, so analyses were collapsed across sex. Fig. 2B shows significant main effects of odor (F(1, 14) = 29.77, p < 0.001) and time (F(7, 98) = 21.79, p < 0.001), and a significant interaction between odor and time (F(7, 98) = 23.55, p < 0.001).

The change from baseline temperature in each rat was analyzed to account for individual differences in transponder-measured baseline temperatures, where there was no significant decrement in thermogenic induction with iBATX. Fig. 2C shows significant main effects of odor (F(1, 14) = 29.77, p < 0.001) and time (F(7, 98) = 21.79, p < 0.001) and a significant interaction between odor and time (F(7, 98) = 23.55, p < 0.001), with no significant interactions between iBATX and odor (F(1, 14) = 0.221, p =0.646), iBATX and time (F(7, 98) = 1.53, p = 0.165), or iBATX, odor, and time (F(7, 98) = 0.694, p = 0.677). Predator odor exposure significantly increased muscle temperature change from baseline relative to control odor for rats with iBATX beginning at 5 min (t(7) = 4.16, p = 0.004) and remaining elevated through 90 min (t(7) = 4.99, p = 0.002), and for rats with intact iBAT (sham) beginning at 10 min (t(7) = 2.99, p = 0.02) and remained elevated through 90 min (t(7) = 3.90, p = 0.006). A repeated measures ANOVA showed no significant interaction between odor and sex (F(1, 12) = 1.099, p = 0.089) and no other significant sex interactions, so all analyses were collapsed across sex.

To control for the potential contribution of contractile thermogenesis secondary to differences in physical activity, locomotion was controlled using constant treadmill walking (Fig. 2D). As shown in Fig. 2E, treadmill walking induced significant muscle thermogenesis in rats with iBATX similar to intact rats. Post-hoc analyses showed that rats lacking iBAT significantly increased muscle temperature after predator odor compared to control odor exposure beginning at 15 min (t(7) = 4.62, p = 0.002) through 35 min (t(7) = 4.58, p = 0.003); rats with intact iBAT (sham) showed the same significant increase in predator odor-induced muscle temperature at 15 min (t(7) = 2.43, p = 0.045) through 35 min (t(7) = 5.46, p = 0.001). The three-way repeated measures ANOVA showed significant main effects of odor (F(1, 14) = 39.80, p < 0.001) and time (F(8, 112) = 68.53, p < 0.001), and a significant interaction between odor and time (F(8, 112) = 10.31, p < 0.001), and no significant interactions between iBATX and odor (F(1, 14) = 0.918, p = 0.354), iBATX and time (F(8, 112) = 1.18, p = 0.316), or iBATX, odor, and time (F(8, 112) = 0.231, p = 0.984). A repeated measures ANOVA showed no significant interaction between odor and sex (F(1, 12) = 2.24, p = 0.160) and no other significant sex interactions, so all analyses were collapsed across sex.

During treadmill walking, muscle temperature increase from baseline did not differ with iBATX, as shown in Fig. 2F. Post-hoc paired samples *t*-tests show that, relative to control-odor exposure, predator odor significantly increased muscle temperature change from baseline beginning at 20 min through 35 min in both rats lacking iBAT (20 min: t(7) = 3.04, p = 0.019; 35 min: (t(7) = 2.53, p = 0.013)) and rats with intact BAT (20 min: t(7) = 3.15, p = 0.016; 35 min: t(7) = 3.60, p = 0.009). A three-way repeated measures ANOVA showed significant main effects of odor (F(1, 14) = 9.86, p = 0.007) and time (F(7, 98) = 18.37, p < 0.001), and a significant interaction between odor and time (F(7, 98) = 14.50, p *<* 0.001), with no significant interactions between iBATX and odor (F(1, 14) = 0.231, p = 0.638), iBATX and time (F(7, 98) = 1.46, p = 0.190), or iBATX, odor, and time (F(7, 98) = 0.220, p = 0.980). The three-way repeated measures ANOVA showed no significant interaction between odor and sex (F(1, 12) = 0.029, p = 0.868) and no other significant sex interactions, so all analyses were collapsed across sex.

In summary, predator odor significantly increased muscle thermogenesis in rats lacking iBATX, with no decrement relative to rats with intact BAT. Predator odor significantly increased skeletal muscle temperature beyond the level of contractile thermogenesis, with no significant impact of iBATX (Fig. 2E & F). Overall, rats with iBATX were able to show the full range of muscle thermogenic induction induced by both activity and predator threat, reaching significantly elevated temperatures earlier than sham-control rats (Fig. 2B).

### 3.3. Surgical excision of iBAT did not significantly alter predator odor-induced changes in energy expenditure or respiratory exchange ratio

Energy expenditure and substrate utilization were monitored using respirometry to assess the impact of iBAT removal on metabolic outcomes—EE, RER, or locomotion—in the context of predator threat (Fig. 3A). A three-way repeated measures ANOVA showed a significant effect of sex on EE (F(1, 14) = 12.48, p = 0.003); as expected due to their size disparity, EE was elevated in males relative to females. Therefore, further analyses were conducted separately for males and females, which both showed significant increase in EE after exposure to predator odor, but no difference with iBATX. As shown in Fig. 3B, male rats showed no significant effects of iBATX on EE or the energetic response to predator odor. A three-way repeated measures ANOVA showed significant main effects of odor (F(1, 6) = 8.50, p = 0.027) and time (F(3, 18) = 14.25, p *<* 0.001). Post-hoc analyses revealed that predator odor exposure significantly increased EE relative to control odor beginning at the time of predator odor presentation (t(7) = 2.42, p = 0.046) and remaining through 2 h (t(7) = 2.58, p = 0.036). Similarly, female rats also showed significant main effects of odor (F(1, 6) = 54.50, p *<* 0.001) and time (F(3, 18) = 19.26, p *<* 0.001) on EE, with a significant interaction between odor and time (F(3, 18) = 4.20, p = 0.02). As with males, there were no significant effects of iBATX. Post-hoc comparisons showed that predator odor exposure significantly increased EE relative to control odor beginning at the time of predator odor presentation (t(7) = 2.30, p = 0.05) and remaining through 3 h (t(7) = 3.24, p = 0.014).

As shown in Fig. 3C, for RER, a three-way repeated measures ANOVA showed a significant interaction between odor and sex (F(1, 12) = 4.28, p = 0.05), so further analyses were conducted separately for males and females. For male rats, a three-way repeated measures ANOVA showed significant main effects of odor (F(1, 6) = 6.03, p = 0.049) and time (F(3, 18) = 13.99, p < 0.001), and a significant interaction between odor and time (F(3, 18) = 7.02, p = 0.013) where RER was lower during predatorodor exposure than control-odor exposure, with no significant effects of iBATX. Post-hoc paired samples *t*-tests showed predator odor exposure significantly decreased RER relative to control odor beginning after 1 h of predator odor exposure (t(7) = −2.42, p = 0.046) and remaining through 2 h (t(7) = −3.71, p = 0.008). For female rats, a three-way repeated measures ANOVA showed no significant main effect of odor (F(1, 6) = 1.68, p = 0.243), a significant main effect of time (F(3, 18) = 12.70, p < 0.001), and a significant interaction between odor and time (F(3, 18) = 4.60, p = 0.015) where RER was higher during predator odor than control exposure. Again, there were no significant effects of iBATX. Post-hoc comparisons between predator odor and control odor showed predator odor exposure significantly increased RER relative to control odor beginning at the time of predator odor exposure (t(7) = 3.00, p = 0.02) and remaining through the first hour of exposure. There was no significant difference in RER after the first hour of exposure.

Both iBATX and predator odor exposure significantly affected ambulatory activity (Fig. 3D). A three-way repeated measures ANOVA showed a significant effect of sex (F(1, 14) = 5.34, p = 0.037), so further analyses were conducted separately for males and females. For male rats, a three-way repeated measures ANOVA showed significant main effects of odor (F(1, 6) = 71.20, p *<* 0.001) and time after odor exposure (F(3, 18) = 31.06, p *<* 0.001), along with a significant interaction between odor and time (F(3, 18) = 5.38, p = 0.008). For ambulatory activity, there was a significant main effect of iBATX (F(1, 6) = 8.61, p = 0.026) and significant interactions between odor and iBATX (F(1, 6) = 15.00, p = 0.008) and between time and iBATX (F(3, 18) = 3.59, p = 0.034) where iBATX rats had higher locomotion compared to sham controls; predator odor exposure significantly increased locomotion relative to control odor beginning at the time of predator odor presentation (t(7) = 3.41, p = 0.011) and remaining for the first 1 h of exposure (t(7) = 2.81, p = 0.026), but separate analyses indicated that this was significant for rats with iBATX only (t(6) = 2.37, p = 0.05). For females, a three-way repeated measures ANOVA showed significant main effects of odor (F(1, 6) = 18.76, p = 0.005) and time (F(3, 18) = 116.54, p < 0.001), and a significant interaction between odor and time (F(3, 18) = 3.31, p = 0.044) with no significant effects of iBATX. Post-hoc paired samples *t*-tests between predator and control odors show predator odor exposure significantly increased locomotion relative to control odor during the first 1 h of exposure (t(7) = 4.89, p = 0.002). Paired samples t-tests showed no significant differences in body weight for predator odor and control odor exposure conditions between treatments for sham or iBATX rats within either male or female rats.

### 3.4. β3-AR agonist differentially alters RER with BATX

To determine if excision of iBAT was sufficient to produce significant metabolic changes, EE, RER, and locomotion were measured following pharmacological activation of BAT with the β3-AR agonist CL-316,243 during indirect calorimetry (Fig. 4A). Briefly, the β3-AR agonist significantly increased EE, and iBATX suppressed EE in female but not male rats. As shown in Fig. 4B, a three-way repeated measures ANOVA showed a significant effect of sex on EE (F(1, 12) = 56.73, p *<* 0.001), so further analyses were conducted separately for males and females. For males, a three-way repeated measures ANOVA showed no significant effects of iBATX. There was a significant main effect where EE increased in response to the β3-AR agonist CL-316,243 compared to vehicle (F(1, 6) = 66.41, p *<* 0.001), a main effect of time (F(7, 42) = 6.09, p *<* 0.001), and a significant interaction between treatment and time (F(7, 42) = 3.88, p = 0.002). Post-hoc comparisons showed that the β3-AR agonist significantly increased EE relative to vehicle beginning at 1 h after injection (t(7) = 10.61, p *<* 0.001) and remaining through 6 h (t(7) = 6.17, p *<* 0.001). For female rats, a three-way repeated measures ANOVA showed a significant main effect where the β3-AR agonist increased EE (F(1, 6) = 31.96, p = 0.001), a main effect of time (F(7, 42) = 13.80, p *<* 0.001), and a significant interaction between treatment and time (F(7, 42) = 7.42, p *<* 0.001). Post-hoc comparisons indicated that the β3-AR agonist significantly increased EE relative to vehicle beginning at 1 h after injection (t(7) = 5.31, p = 0.001) through 8 h (t(7) = 3.00, p = 0.02). Comparing EE after treatment with the β3-AR agonist CL-316,243, the ability of the β3-AR agonist to elevate EE was lower in female BATX rats relative to sham-control females, reaching significance at one time point (3 h; unpaired *t*-test; Fig. 4B).

As shown in Fig. 4C, the β3-AR agonist CL-316,243 also lowered RER, and this effect differed in rats with iBATX. A three-way repeated measures ANOVA collapsed across sex showed a significant 3-way interaction between treatment, time, and iBATX (F(7, 98) = 3.38, p = 0.003) where the decrease in RER with the β3-AR agonist differed between rats with iBATX compared to the sham procedure. A three-way repeated measures ANOVA showed a significant interaction between treatment and sex (F(1, 12) = 10.85, p = 0.006), so further analyses were conducted separately for males and females. For male rats, a threeway repeated-measures ANOVA showed significant main effects of treatment (F(1, 6) = 156.84, p *<* 0.001) and time (F(7, 42) = 31.14, p *<* 0.001), as well as significant interactions between treatment and time (F (7, 42) = 8.59, p *<* 0.001), and between treatment, time, and iBATX (F (7, 42) = 3.69, p = 0.003). Post-hoc paired samples *t*-tests between β3- AR agonist and vehicle show the β3-AR agonist significantly decreased RER relative to vehicle beginning at 1 h after injection (t(7) = −12.06, p < 0.001) and remaining through 8 h (t(7) = −8.58, p *<* 0.001). In female rats, RER showed a significant main effects of treatment (F(1, 6) = 8.36, p = 0.028) and time (F(7, 42) = 24.97, p *<* 0.001), and an interaction between treatment and time (F(7, 42) = 3.69, p = 0.003). Post-hoc comparisons showed that the β3-AR agonist significantly decreased RER relative to vehicle beginning at 1 h after injection (t(7) = −4.88, p = 0.002) and remaining through 3 h (t(7) = −3.09, p = 0.017).

Physical activity was not significantly altered by either the β3-AR agonist nor iBATX. A three-way repeated measures ANOVA showed no significant effect of sex (F(1, 12) = 1.56, p = 0.235), so further analyses were collapsed across sex. A three-way repeated measures ANOVA showed no significant effects of treatment (F(1, 14) = 1.14, p = 0.303), time (F(1, 14) = 3.28, p =0.066), or surgery (F(1, 14) = 2.63, p = 0.127) on physical activity, and no significant interactions (Fig. 4D).

Paired samples t-tests showed no significant differences in male body weights between treatments for sham or iBATX rats (t(3) = −1.59, p = 0.211; t(3) = −0.670, p = 0.193) or between BATX compared to sham surgery for agonist or vehicle treatments (t(3) = 0.407, p = 0.711; t(3) = 0.381, p = 0.729). Similarly, there were no significant differences in female body weights between treatments for sham or iBATX rats (t(3) = 1.44, p = 0.245; t(3) = 1.47, p = 0.238) or between surgery type for agonist or vehicle treatments (t(3) = 1.70, p = 0.189; t(3) = 0.538, p = 0.628).

## 4. Discussion

Consistent with prior evidence (Gorrell et al., 2020; Shemery et al., 2025, 2023), here we report that exposure to predator threat in the form of ferret odor induced a marked, rapid increase in muscle temperature in rats (Fig. 2B) (Gorrell et al., 2020; Shemery et al., 2023). Exposure to ferret odor increased SNS outflow to skeletal muscle (Fig. 1B), consistent with previous evidence supporting the importance of SNS neural communication with muscle in this process (Gorrell et al., 2020). Sympathetic outflow was also increased to both white and brown adipose tissue after exposure to predator threat (Fig. 1C & E), presenting another potential source of heat. Here, we found that surgical removal of the primary BAT depot, iBAT, did not impair muscle thermogenesis (Fig. 2). Removal of iBAT did not significantly alter the ability of predator threat to increase EE (Fig. 3B). Some alteration in the ability of the β3 adrenergic agonist CL-316,243 or predator odor to alter EE, substrate utilization, or locomotor activity was evident, though (Figs. 3 & 4). Altogether, these findings reinforce the importance of direct neural SNS outflow to skeletal muscle in the thermogenic response to predator threat.

Assessment of NETO rate is an established indicator of SNS outflow to multiple organs simultaneously (Vaughan et al., 2014), and we have previously demonstrated that induction of muscle thermogenesis with central melanocortin receptor activation also increases SNS drive to skeletal muscle (Gavini et al., 2016). Here, we show that exposure to predator threat in the form of ferret odor elevated SNS outflow to skeletal muscles (Fig. 1B). This is consistent with the marked suppression in thermogenesis following unilateral SNS denervation as well as the ability of the peripherally acting beta-blocker nadolol to inhibit thermogenesis (Gorrell et al., 2020). Elevated NETO after predator-odor exposure was seen in oxidative, glycolytic, and mixed muscle of the hindlimb, with no noticeable bias toward muscle type (Fig. 1B). The lumbar sympathetic nerve (LSN) serves the muscles of the hindlimb, with differing numbers of ganglia supplying each muscle (Peyronnard et al., 1986); innervation level does not appear to correspond to the magnitude of SNS drive after thermogenic stimulation, similar to prior investigation of muscle NETO after brain stimulation of melanocortin receptors (Gavini et al., 2018, 2016). The response to SNS outflow within muscle groups may differ based on muscle fiber type (e.g., oxidative, glycolytic, or mixed) (Zierath and Hawley, 2004), as the density of β-ARs varies among muscle subgroups (Williams et al., 1984).

Sympathetic outflow to organs outside muscle, including heart, liver, and adipose tissue (Bartness et al., 2010a, 2010b; Ryu et al., 2015; Schmelck and Hanoune, 1980; Xiao et al., 2006) also contributes to metabolic and thermogenic processes (Abumrad, 2017; Carpentier et al., 2018; Hiilloskorpi et al., 2003; Saito, 2013; Shiota et al., 1992; Smorlesi et al., 2012; Stoner, 1973; Townsend and Wright, 2019; Zoladz et al., 2013). SNS activation of these alternate thermogenic targets may contribute to the thermogenesis seen after predator odor exposure or support the ability of muscle to respond to predator threat. Here, elevated NETO rate was also seen in BAT after predator threat (Fig. 1C), which likely drives the parallel thermogenesis seen in BAT after exposure to ferret odor (Gorrell et al., 2020). There was no significant elevation in NETO rate detected in either heart or liver (Fig. 1D), however, despite the survival value of increased heart rate and metabolic fuel allocation from the liver (Karnani and Burdakov, 2011; Yi et al., 2010). Alternatively, SNS stimulation of WAT lipolysis could also provide fuel in the form of free fatty acids for the skeletal muscle, serving the energetic demands required for thermogenesis. A significant increase in NETO was detected in three WAT depots—eWAT, gWAT, and rWAT—but not in iWAT or mWAT (Fig. 1E). The importance of SNS stimulation of WAT lipolysis and body fat (Youngstrom and Bartness, 1998; Zeng et al., 2015) raises the question of the potential for increased myocyte uptake of metabolic fuels, including fatty acids, after exposure to predator threat. Given that ferret-odor exposure does not consistently increase or decrease RER here (Fig. 3C) compared to previous findings (Gorrell et al., 2020), it is likely that both glucose and fatty acid oxidation contribute to the elevated caloric expenditure of muscle. The potential for predator-odor exposure to promote WAT browning has not been assessed, though the acute elevation in sympathetic outflow evidenced after a single encounter with predator threat is unlikely to be sufficient to promote beige WAT or acutely increase WAT thermogenesis or body temperature to the extent seen in rats presented with predator odor (Gorrell et al., 2020; Shemery et al., 2023).

The ability of predator threat to increase SNS outflow to BAT (Fig. 1C) and BAT thermogenesis (Gorrell et al., 2020) suggests the possibility that this same stimulus may increase factors secreted from BAT called batokines; these could impact muscle, including its thermogenesis (Gaspar et al., 2021; Ortiz and de Freitas, 2023; Villarroya et al., 2017). It is also possible that increased body temperature stemming from BAT thermogenesis could increase muscle temperature. To examine the potential contribution of BAT to skeletal muscle thermogenesis in the context of predator threat, we excised the major BAT depot, iBAT, in rats and measured the thermogenic response to ferret odor. As shown in Fig. 2, exposure to ferret odor significantly elevated muscle temperature in rats with intact BAT as well as in rats with iBAT excised. There was no discernable decrement in muscle temperature secondary to iBATX in freely moving rats (Fig. 2B & C). Given the ability of predator-odor exposure to increase locomotion (see Fig. 3D), which could potentially confound the measurement of muscle temperature, muscle thermogenic response to ferret odor was assessed in iBATX and sham-control rats while physical activity levels were fixed using treadmill walking (see Fig. 2A). As shown in Fig. 2D & E, walking at 7 m/min on a treadmill increased muscle temperature, and the elevation in temperature was significantly enhanced after exposure to predator odor compared to the control odor, consistent with prior evidence (Gorrell et al., 2020). Both treadmill walking and exposure to ferret odor elevated muscle temperature similarly in iBATX rats and sham-operated control rats (Fig. 2D & E). Altogether, the lack of iBAT did not cause any discernable decrement in the ability of predator threat to provoke muscle thermogenesis in rats.

The elevation in muscle temperature with predator-odor exposure was accompanied by a marked increase in EE (Fig. 3B), consistent with previous reports (Gorrell et al., 2020). Though predator odor exposure increased temperature of BAT alongside muscle (Gorrell et al., 2020), removal of iBAT had no significant effect on metabolic rate in either male or female rats, with or without contextual thermogenic induction (Fig. 3B), implicating muscle thermogenic processes as the primary source of caloric expenditure during ferret odor encounter. Compared to control odor, exposure to ferret odor significantly increased locomotor activity in both male and female rats (Fig. 3D), consistent with prior findings (Gorrell et al., 2020). Interestingly, iBAT removal influenced activity levels in male rats (Fig. 3D). While predator odor-induced increases in ambulation could elevate muscle temperature through contractile thermogenesis, ferret odor significantly increased muscle temperature even when ambulation was held constant using treadmill walking (Fig. 2D–F), in line with prior reports (Gorrell et al., 2020). With respect to metabolic and thermogenic processes, it is possible that rats adapted to the surgical excision of BAT akin to what occurs after lipectomy of white adipose tissue (Mauer et al., 2001), potentially compensating for lower BAT thermogenesis by elevating thermogenesis and EE in other BAT depots, muscle, or even through elevated physical activity (see Fig. 3D). That said, our finding that the ability of predator threat to elevate both muscle temperature (Fig. 2) and metabolic rate (Fig. 3) persists in the absence of BAT is consistent with evidence of stress-induced hyperthermia in response to conditioned fear even without concomitant β-AR-mediated BAT thermogenesis (Marks et al., 2009).

The β3-AR agonist CL-316,243 was used to assess the impact of iBAT removal (Fig. 4A). Modulation of the metabolic effects of CL-316,243 by iBAT excision were moderate. In female rats, the ability of CL-316,243 to increase EE differed with iBAT excision, where sham-operated control rats showed higher EE (Fig. 4B). In male rats, RER differed between rats with iBAT excision compared to sham controls after CL-316,243 treatment (Fig. 4C). Given these modest impacts of BAT excision on metabolic outcomes, it is possible that other BAT depots contributed to the ability of CL-316,243 to alter EE and RER. For example, BAT depots in the neck and shoulders appear to be relevant in humans (Richard and Picard, 2011). β3-AR may also have thermogenic actions in other tissues, including white adipocytes (Dongdem et al., 2024; Natarajan et al., 2024), and could affect muscle metabolic pathways as well as muscle function (Ding et al., 2021; Puzzo et al., 2016; Straat et al., 2023). Lastly, while β3-AR agonism with CL-316,243 had a marked effect on substrate utilization, with the lower RER (Fig. 4C) indicating relative increase in lipid oxidation, changes in RER with iBATX and predator odor exposure (Fig. 3C) were less distinct and less consistent with prior reports (Gorrell et al., 2020).

The evidence presented here also reinforces the lack of a sex difference in muscle thermogenesis and the response to predator threat, despite sex differences in body size and metabolic control between males and females. We have previously shown that predator odor-induced thermogenesis did not differ between the proestrus and diestrus phases of the estrous cycle in female rats (Gorrell et al., 2020). In fact, the only sex difference noted regarding muscle thermogenesis reported by Gorrell et al. (2020) was the greater thermogenic reaction to the experimental context, which was eliminated by adequate habituation; this divergence likely stems from sex differences in the locomotor and endocrine response to novelty and adaptation to acute stressors (reviewed by Novak and Levine (2007), Oyola and Handa (2017), and Rincon-Cortes et al. (2019)). Sex differences in gas exchange parameters primarily result from the much larger body size of male rats compared to female rats, combined with known effects of sex and gonadal hormones on metabolic control. The male rats have much higher EE than females, both at baseline and after exposure to ferret odor (Fig. 3B). Interestingly, the change in fuel allocation, reflected in RER, differed between male and female rats under baseline (vehicle control) conditions (Fig. 4C). Altogether, there were no major sex differences in the thermogenic or energetic response to predator threat.

Taken together with prior evidence (Gorrell et al., 2020), the data reported here reinforce the importance of direct neural SNS outflow to the ability of predator threat to stimulate muscle thermogenesis. Increased SNS drive to WAT after predator threat may stimulate lipolysis and elevate fatty acid mobilization to fuel muscle contraction. Elevated SNS drive to BAT is also evident (Fig. 1C), which is likely stimulating the increased BAT temperature seen after predator odor exposure (Gorrell et al., 2020). As iBAT removal does not significantly impact muscle temperature (Fig. 2), the muscle thermogenic response to ferret odor appears to stem from the SNS neural stimulation of muscles directly, rather than secondary to BAT.

## Data Availability

Data will be made available on request.

## Abbreviations:

ANOVA: analysis of variance
AMPT: alpha-methyl-p-tyrosine
BAT: brown adipose tissue
β2-ARs: beta-2 adrenergic receptor
β3-ARs: beta-3 adrenergic receptor
EE: energy expenditure
eWAT: epididymal white adipose tissue
EDL: extensor digitorum longus
gWAT: gluteal white adipose tissue
HPLC analysis: high performance liquid chromatography
iBAT: interscapular BAT
iBATX: iBAT excision
iWAT: inguinal white adipose tissue
LPM: liters per minute
mWAT: mesenteric white adipose tissue
NETO: norepinephrine turnover
RER: respiratory exchange ratio
rWAT: retroperitoneal white adipose tissue
SNS: sympathetic nervous system
WAT: white adipose tissue
